# Total-Body Multiparametric PET Quantification of ^18^F-FDG Delivery and Metabolism in the Study of Coronavirus Disease 2019 Recovery

**DOI:** 10.2967/jnumed.123.265723

**Published:** 2023-11

**Authors:** Yiran Wang, Lorenzo Nardo, Benjamin A. Spencer, Yasser G. Abdelhafez, Elizabeth J. Li, Negar Omidvari, Abhijit J. Chaudhari, Ramsey D. Badawi, Terry Jones, Simon R. Cherry, Guobao Wang

**Affiliations:** 1 Department of Radiology, Davis Medical Center, University of California, Sacramento, California;; 2 Department of Biomedical Engineering, University of California, Davis, Davis, California; and; 3 Nuclear Medicine Unit, South Egypt Cancer Institute, Assiut University, Assiut, Egypt

**Keywords:** ^18^F-FDG PET, tracer kinetic modeling, total-body dynamic PET, COVID-19

## Abstract

Conventional whole-body static ^18^F-FDG PET imaging provides a semiquantitative evaluation of overall glucose metabolism without insight into the specific transport and metabolic steps. Here we demonstrate the ability of total-body multiparametric ^18^F-FDG PET to quantitatively evaluate glucose metabolism using macroparametric quantification and assess specific glucose delivery and phosphorylation processes using microparametric quantification for studying recovery from coronavirus disease 2019 (COVID-19). **Methods:** The study included 13 healthy subjects and 12 recovering COVID-19 subjects within 8 wk of confirmed diagnosis. Each subject had a 1-h dynamic ^18^F-FDG scan on the uEXPLORER total-body PET/CT system. Semiquantitative SUV and the SUV ratio relative to blood (SUVR) were calculated for different organs to measure glucose utilization. Tracer kinetic modeling was performed to quantify the microparametric blood-to-tissue ^18^F-FDG delivery rate $K_{1}$ and the phosphorylation rate *k*_3_, as well as the macroparametric ^18^F-FDG net influx rate ($K_{\text{i}}$). Statistical tests were performed to examine differences between healthy subjects and recovering COVID-19 subjects. The effect of COVID-19 vaccination was also investigated. **Results:** We detected no significant difference in lung SUV but significantly higher lung SUVR and $K_{\text{i}}$ in COVID-19 recovery, indicating improved sensitivity of kinetic quantification for detecting the difference in glucose metabolism. A significant difference was also observed in the lungs with the phosphorylation rate *k*_3_ but not with $K_{1}$, which suggests that glucose phosphorylation, rather than glucose delivery, drives the observed difference of glucose metabolism. Meanwhile, there was no or little difference in bone marrow ^18^F-FDG metabolism measured with SUV, SUVR, and $K_{\text{i}}$ but a significantly higher bone marrow $K_{1}$ in the COVID-19 group, suggesting a difference in glucose delivery. Vaccinated COVID-19 subjects had a lower lung $K_{\text{i}}$ and a higher spleen $K_{\text{i}}$ than unvaccinated COVID-19 subjects. **Conclusion:** Higher lung glucose metabolism and bone marrow glucose delivery were observed with total-body multiparametric ^18^F-FDG PET in recovering COVID-19 subjects than in healthy subjects, implying continued inflammation during recovery. Vaccination demonstrated potential protection effects. Total-body multiparametric PET of ^18^F-FDG can provide a more sensitive tool and more insights than conventional whole-body static ^18^F-FDG imaging to evaluate metabolic changes in systemic diseases such as COVID-19.

## Visual Abstract

**Figure:**
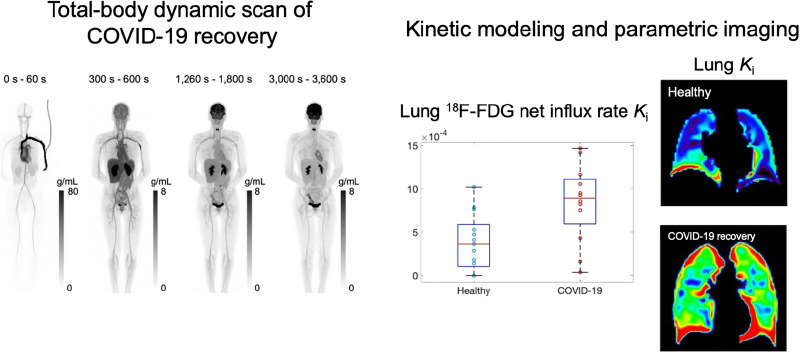

PET with the radiotracer ^18^F-FDG is a noninvasive in vivo molecular imaging technique that reflects glucose metabolism. Conventional whole-body static ^18^F-FDG PET imaging can provide an overall evaluation of glucose utilization throughout the body, but it mixes the specific glucose transport and metabolic steps. Identification and quantification of these specific processes separately require a fast dynamic scanning protocol; however, it is limited to a single organ or a confined region by a PET scanner with a short axial field of view. The advent of total-body PET/CT systems such as uEXPLORER (United Imaging Healthcare) (1) and other PET scanners with a long axial field of view (2,3) has brought new opportunities for total-body dynamic PET imaging, with increased detection sensitivity and simultaneous dynamic imaging of multiple organs (4). Combined with tracer kinetic modeling (5), total-body dynamic ^18^F-FDG PET enables a multiparametric quantification method (6) that allows quantitative measurement of not only overall glucose utilization but also microparametric rates of glucose delivery and phosphorylation (7) over the entire body.

Although mostly used in oncology, ^18^F-FDG PET has the potential to characterize inflammatory diseases such as vasculitis (8), hepatitis (9), osteomyelitis (10), and the recent coronavirus disease 2019 (COVID-19) (11–14). COVID-19 primarily attacks the respiratory system, leading to conditions varying from mild manifestations to acute, high-mortality symptoms (15). Meanwhile, it can affect multiple organs associated with different body systems, including the nervous (16), cardiovascular (17), and immune systems (18). In addition, various prolonged effects of COVID-19 have been reported (19–22). However, investigations of the whole-body consequences and prolonged effects from COVID-19 are limited, partially because of the lack of an approach for in-depth total-body evaluation.

For this article, we conducted a quantitative evaluation of glucose utilization in multiple organs of healthy subjects and recovering COVID-19 subjects using total-body multiparametric ^18^F-FDG PET imaging. We analyzed overall glucose metabolism and, more subtly, the blood-to-tissue glucose delivery and glucose phosphorylation to gain insight into the metabolic differences induced by COVID-19.

## MATERIALS AND METHODS

### Study Participants and Data Acquisition

With Institutional Review Board approval and written informed consent at University of California Davis Health, the study included a cohort of 13 healthy subjects and 12 COVID-19 subjects. The healthy subjects were scanned between May 2019 and January 2020. They had no history of major disease (e.g., cancer or myocardium infarction) over the previous 5 y and lacked ongoing acute inflammation. The COVID-19 subjects had mild to moderate symptoms, as summarized in Supplemental Table 1 (supplemental materials are available at http://jnm.snmjournals.org), and none of them were hospitalized. Seven COVID-19 subjects had 1–3 doses of COVID-19 vaccines before PET imaging, and the other 5 were not vaccinated. Each subject had a total-body 1-h ^18^F-FDG dynamic scan on the uEXPLORER PET/CT system (23,24). The PET/CT scans for the COVID-19 subjects were performed within 8 wk (37 ± 16 d) of confirmed diagnosis. All COVID-19 subjects tested negative for COVID-19 11 ± 7 d before the PET scan (inclusion and exclusion criteria are summarized in the supplemental materials). The subjects were injected with 333 ± 45 MBq of ^18^F-FDG intravenously immediately after initiating list-mode data acquisition. A total-body ultra–low-dose CT scan with settings of 140 kVp and 5 mAs was performed before the PET scan for attenuation correction. Dynamic PET data were reconstructed into 29 frames (6 × 10 s, 2 × 30 s, 6 × 60 s, 5 × 120 s, 4 × 180 s, and 6 × 300 s) with a voxel size of 4 × 4 × 4 mm^3^ using the vendor-provided ordered-subset expectation maximization algorithm with 4 iterations and 20 subsets (23).

### Total-Body Kinetic Modeling

Regions of interest (ROIs) were placed in various organs and tissues (e.g., brain, liver, lungs, spleen, and bone marrow) throughout the entire body on the dynamic images of each subject (details of ROI placement are in Supplemental Table 2 and Supplemental Fig. 1). Time–activity curves were then extracted from the organ ROIs. In addition, ROI placement and time–activity curve extraction were done for the ascending aorta and right ventricle to acquire image-derived input functions.

A 2-tissue irreversible compartmental model, shown in Supplemental Figure 2, was used to model the dynamic ^18^F-FDG data with time delay correction included (6). The measured tissue time–activity curve $C_{\text{T}}\left(t\right)$ was modeled as follows:

$$C_{\text{T}}\left(t\right)=\left(1-v_{\text{b}}\right)\left(C_{\text{f}}\left(t\right)+C_{\text{m}}\left(t\right)\right)+v_{\text{b}}C_{\text{wb}}\left(t\right),$$ Eq. 1

where $C_{\text{wb}}\left(t\right)$, $C_{\text{f}}\left(t\right)$, and $C_{\text{m}}\left(t\right)$ represent the concentrations of whole blood ^18^F-FDG, tissue free-state ^18^F-FDG, and tissue-metabolized ^18^F-FDG-6P, respectively, and $v_{\text{b}}$ is the fractional blood volume. Details of the compartmental model are described in the supplemental materials.

All kinetic parameters ($K_{1}$, blood-to-tissue ^18^F-FDG delivery rate; $k_{2}$, tissue-to-blood delivery rate; and $k_{3}$, ^18^F-FDG phosphorylation rate, fractional blood volume *v*_b_, and the time delay for input function *t*_d_) were jointly estimated through a nonlinear least-square fitting method (6) with a weighting factor that considers the time length of each frame and nuclear decay (25).

### Macroparametric and Microparametric Quantification

The macroparameter *K*_i_, denoting the ^18^F-FDG net influx rate, is commonly used to characterize overall glucose metabolism and is calculated as follows:

$$K_{\text{i}}=\frac{K_{1}k_{3}}{k_{2}+k_{3}}.$$ Eq. 2

We also applied semiquantitative SUV (26) and the SUV ratio relative to blood (SUVR) (27) using the last dynamic frame (55–60 min) to evaluate overall glucose metabolism. As described in the supplemental materials, the right ventricle was used to extract the image-derived input function for the lung SUVR calculation, and the ascending aorta was used for the SUVR calculation of all other organs (28).

In addition to the measures of overall ^18^F-FDG metabolism by SUV, SUVR, and $K_{\text{i}}$, we used the microparameters of the 2-tissue irreversible kinetic model, specifically $K_{1}$ and $k_{3}$, to gain insight into the individual molecular processes of glucose utilization. The ability of this microparametric quantification is a feature that distinguishes compartmental modeling from whole-body static imaging or whole-body dynamic imaging with a simplified graphical analysis method (e.g., the Patlak plot).

### Statistical Analysis

Statistical analysis in this study was performed using an unpaired, 2-tailed *t* test and the Mann–Whitney *U* test on SUV, SUVR, and parametric PET metrics to investigate metabolic differences in the recovering COVID-19 subjects compared with the healthy subjects. In addition, the tests were performed on lung CT ROI quantitation for complementary information. Effect of vaccination was also investigated when appropriate between the vaccinated and the unvaccinated COVID-19 groups (29,30). All statistical data analyses were conducted using MATLAB (MathWorks). *P* values of less than 0.05 were considered statistically significant.

For organs that showed a trend of differences in glucose metabolism between the healthy and the COVID-19 groups, Pearson correlation analysis and Spearman rank correlation analysis between $K_{\text{i}}$ and microparameters $K_{1}$, $k_{2}$, and $k_{3}$ were also calculated to understand the association among the delivery, phosphorylation, and overall metabolism of ^18^F-FDG.

### Parametric Imaging of COVID-19

In addition to the ROI-based analysis, voxelwise parametric images were generated for the healthy subjects and the recovering COVID-19 subjects using the 2-tissue irreversible compartmental model (31,32). Kernel smoothing was applied to both the dynamic images and the parametric images for noise reduction (6). To focus the comparison of parametric images on organs of interest, masking was used to visualize individual organs or tissues (e.g., lung or bone marrow) within the parametric images for intersubject comparisons.

## RESULTS

### Subject Characteristics

A summary of subject characteristics is provided in Supplemental Table 1. The healthy subjects consisted of 6 men and 7 women of age 49 ± 15 y and weight 82 ± 18 kg. The COVID-19 subjects consisted of 3 men and 9 women of age 41 ± 10 y and weight 84 ± 25 kg. There was no statistical difference between the 2 groups in age, weight, body mass index, blood glucose level, or fasting time before the PET scan using the unpaired *t* test and the *U* test. In addition, there were no statistical differences in lung CT values and in the SUV of the input functions between the 2 groups.

### Dynamic Images and Time–Activity Curves

**FIGURE 1.**
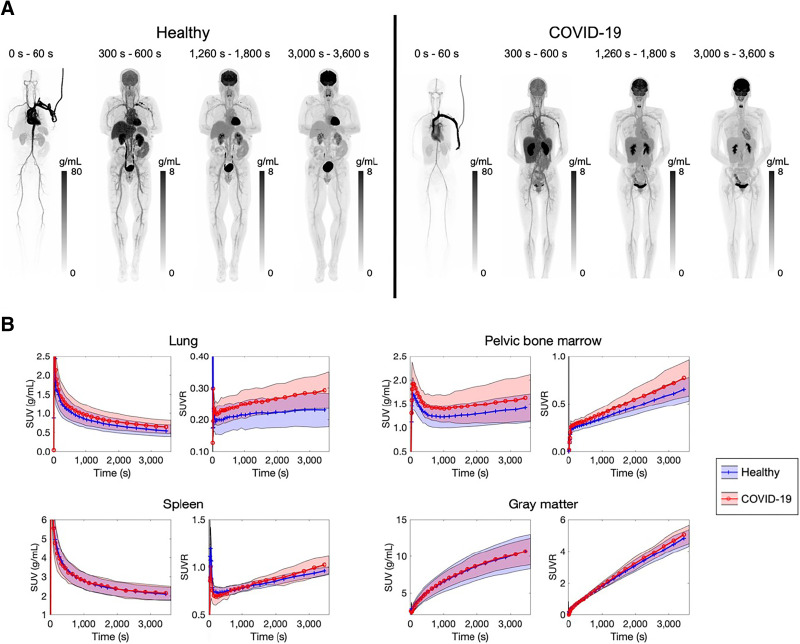
(A) Total-body dynamic ^18^F-FDG PET images of a healthy subject and a recovering COVID-19 subject. Maximum-intensity projections are shown. (B) Averaged time–activity curves (shown as SUV and SUVR) of 4 organs of interest (lung, pelvic bone marrow, spleen, and gray matter) of 13 healthy and 12 recovering COVID-19 subjects. Averaged values are shown as solid lines, and SDs are shown as bands.

Total-body dynamic ^18^F-FDG PET images of a representative healthy subject and a recovering COVID-19 subject are shown in Figure 1A. Figure 1B shows 4 examples of the time–activity curves in the form of SUV and SUVR over time. The most notable finding was the increased lung SUVR in the recovering COVID-19 group compared with the healthy group, whereas the bone marrow SUVR and spleen SUVR of recovering COVID-19 group also tended to be higher.

### Comparison of Overall Glucose Utilization in Multiple Organs

**FIGURE 2.**
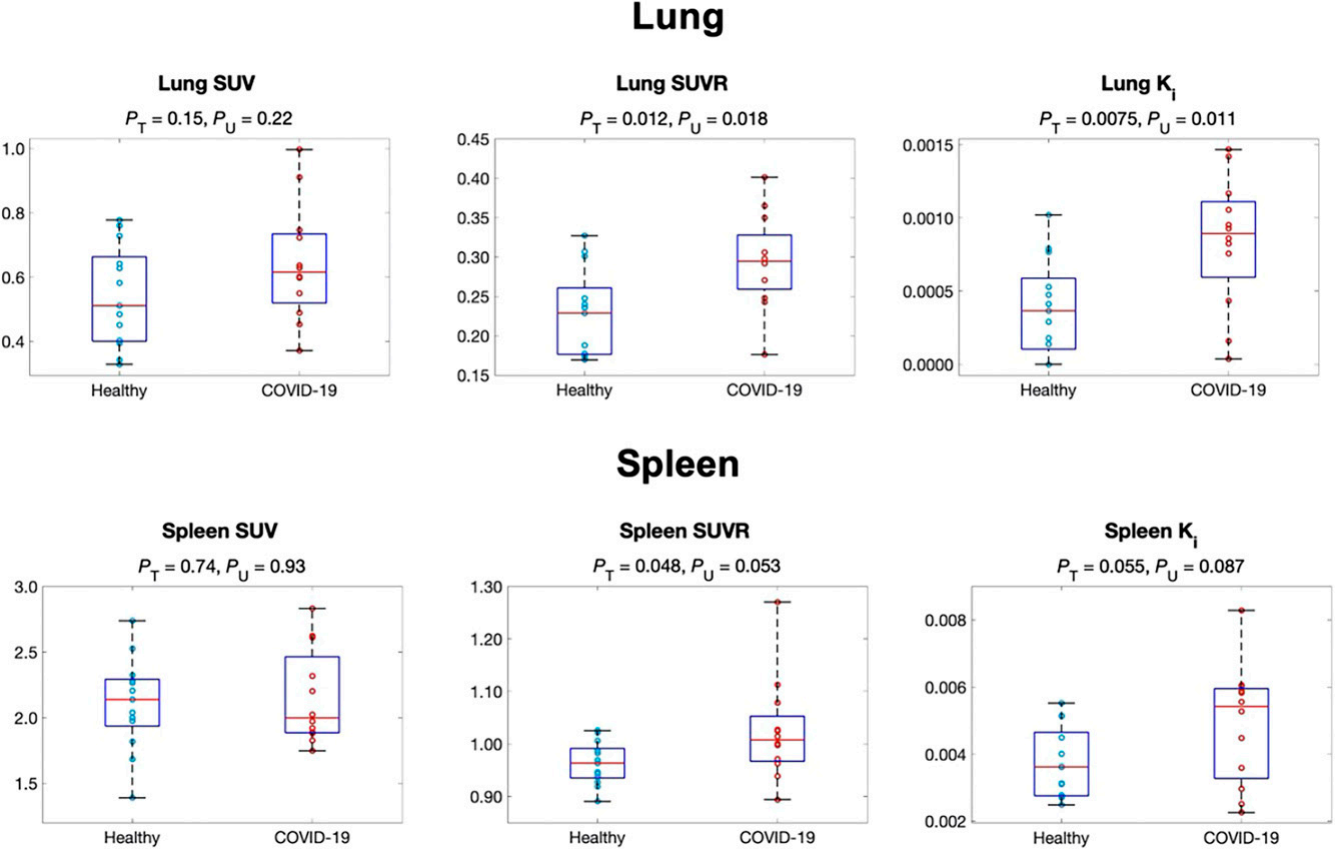
Comparison of ^18^F-FDG metabolism in lung (top) and spleen (bottom) between healthy and recovering COVID-19 groups using SUV, SUVR (both from 55 to 60 min), and $K_{\text{i}}$. *P*_T_ = *P* value of *t* test; *P*_U_ = *P* value of Mann–Whitney *U* test.

Table 1 summarizes the SUV, SUVR, and $K_{\text{i}}$ of the healthy and the recovering COVID-19 groups, along with group comparison results for 11 organ ROIs. There was no significant difference in lung SUV between the 2 groups (*P* > 0.1) (Fig. 2). However, there was a statistically significant increase of approximately 120% in lung $K_{\text{i}}$ in the COVID-19 group (*P* ≈ 0.01). SUVR showed a difference (∼25% increase) but to a lower degree.

**TABLE 1.** Comparison of ^18^F-FDG Metabolic Metrics SUV, SUVR, and $K_{\text{i}}$ Between Healthy Subjects and Recovering COVID-19 Subjects in Multiple Organs and Tissues

Organ or tissue	Metric	Healthy group	Recovering COVID-19 group	*P*_T_	*P*_U_
Lung	SUV	0.54 ± 0.16	0.64 ± 0.18	0.15	0.22
	SUVR	0.230 ± 0.055	0.293 ± 0.060	0.012	0.018
	*K*_i_	0.00038 ± 0.00033	0.00084 ± 0.00045	0.0075	0.011
Myocardium	SUV	7.5 ± 3.5	5.8 ± 2.8	0.21	0.20
	SUVR	3.4 ± 1.6	2.8 ± 1.4	0.38	0.34
	*K*_i_	0.055 ± 0.033	0.043 ± 0.025	0.31	0.37
Liver	SUV	2.64 ± 0.44	2.56 ± 0.40	0.65	0.61
	SUVR	1.208 ± 0.060	1.218 ± 0.061	0.69	0.68
	*K*_i_	0.00279 ± 0.00094	0.00330 ± 0.00086	0.17	0.17
Spleen	SUV	2.11 ± 0.35	2.15 ± 0.36	0.74	0.93
	SUVR	0.963 ± 0.041	1.024 ± 0.097	0.048	0.053
	*K*_i_	0.0037 ± 0.0010	0.0049 ± 0.0018	0.055	0.087
Spine bone marrow	SUV	2.06 ± 0.38	2.21 ± 0.59	0.43	0.57
	SUVR	0.95 ± 0.17	1.05 ± 0.21	0.21	0.22
	*K*_i_	0.0072 ± 0.0015	0.0080 ± 0.0023	0.35	0.50
Pelvic bone marrow	SUV	1.42 ± 0.31	1.63 ± 0.51	0.22	0.43
	SUVR	0.65 ± 0.13	0.77 ± 0.20	0.087	0.13
	*K*_i_	0.0050 ± 0.0012	0.0059 ± 0.0019	0.19	0.24
Thigh muscle	SUV	0.57 ± 0.16	0.58 ± 0.12	0.92	0.93
	SUVR	0.262 ± 0.056	0.279 ± 0.065	0.50	0.72
	*K*_i_	0.00168 ± 0.00057	0.00179 ± 0.00059	0.65	0.89
Gray matter	SUV	10.7 ± 2.4	10.7 ± 1.9	0.99	0.76
	SUVR	4.84 ± 0.54	5.07 ± 0.60	0.33	0.31
	*K*_i_	0.0476 ± 0.0062	0.0487 ± 0.0061	0.65	0.68
White matter	SUV	4.5 ± 1.6	3.9 ± 1.0	0.28	0.22
	SUVR	2.03 ± 0.45	1.85 ± 0.31	0.26	0.46
	*K*_i_	0.0168 ± 0.0051	0.0148 ± 0.0046	0.33	0.50
Brain stem	SUV	6.1 ± 1.3	5.84 ± 0.82	0.55	0.68
	SUVR	2.78 ± 0.24	2.79 ± 0.34	0.90	0.85
	*K*_i_	0.0247 ± 0.0023	0.0241 ± 0.0033	0.62	0.46
Cerebellum	SUV	7.3 ± 1.3	6.99 ± 0.77	0.49	0.50
	SUVR	3.34 ± 0.28	3.35 ± 0.27	0.93	0.89
	*K*_i_	0.0300 ± 0.0033	0.0300 ± 0.0030	1.0	1.0

*P*_T_ = *P* value of *t* test; *P*_U_ = *P* value of Mann–Whitney *U* test.

Groups are mean ± SD, SUV is in g/mL, and *K*_i_ is in mL/min/cm^3^.

The ^18^F-FDG metabolism of the spleen was higher in the COVID-19 group, as shown in Table 1 and the box plots in Figure 2. $K_{\text{i}}$ produced a larger group difference than SUV, whereas SUVR was comparable to $K_{\text{i}}$. The ^18^F-FDG metabolism of the pelvic bone marrow also tended to increase (*P* ≈ 0.1), as shown by the SUVR measures in Table 1 and Supplemental Figure 3. We did not observe a statistically significant difference with SUV, SUVR, and $K_{\text{i}}$ in other organs (e.g., brain and liver).

On the basis of the preceding analyses, the lung, bone marrow, and spleen were selected for further study of microparametric quantification.

### Microparametric Quantification of the Lungs

**FIGURE 3.**
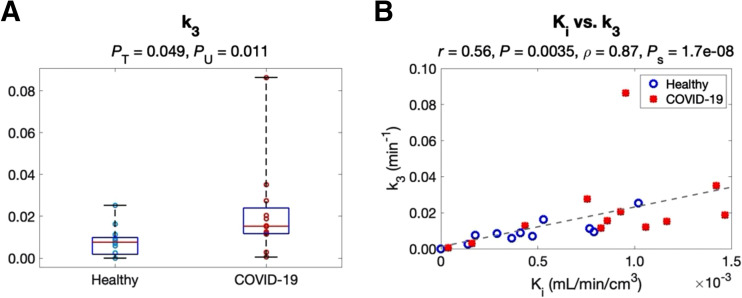
Study of lung kinetic parameters in the healthy and the recovering COVID-19 groups. (A) Comparison of $k_{3}$ between 2 groups. (B) Correlation between $k_{3}$ and $K_{\text{i}}$ among subjects. *P*_S_ = *P* value of Spearman rank correlation; *P*_T_ = *P* value of *t* test; *P*_U_ = *P* value of Mann–Whitney *U* test.

Table 2 shows the analysis of microparametric quantification of the lungs. The correlation between each microparameter and lung $K_{\text{i}}$ is also included using all subject data. Neither $K_{1}$ nor $k_{2}$ detected any group difference (*P* > 0.6). $k_{3}$ was higher in the COVID-19 group (*P* < 0.05), as further shown in Figure 3A. In addition, $k_{3}$ had the strongest correlation with $K_{\text{i}}$ (*P* < 0.01) among the 3 microparameters (Fig. 3B), whereas the correlations of $K_{1}$ and $k_{2}$ with $K_{\text{i}}$ were weaker (*P* > 0.25). The findings suggested that increased ^18^F-FDG phosphorylation (as quantified by $k_{3}$) might be the main driving factor for the increased lung ^18^F-FDG metabolism (assessed by $K_{\text{i}}$) in COVID-19 recovery.

**TABLE 2.** Comparison of Lung Microkinetic Parameters $K_{1}$, $k_{2}$, and $k_{3}$ Between Healthy Subjects and Recovering COVID-19 Subjects, and Correlation Between Microparameters and Lung $K_{\text{i}}$ Using Pearson and Spearman Analyses

Kinetic parameter	Comparison				Correlation with *K*_i_			
	Healthy group	Recovering COVID-19 group	*P*_T_	*P*_U_	Pearson		Spearman	
					*r*	*P*	ρ	*P*_S_
*K*_1_ (mL/min/cm^3^)	0.018 ± 0.022	0.017 ± 0.019	0.89	0.98	0.23	0.26	0.44	0.028
*k*_2_ (min^−1^)	0.32 ± 0.33	0.26 ± 0.25	0.61	0.81	0.17	0.42	0.36	0.075
*k*_3_ (min^−1^)	0.0079 ± 0.0071	0.021 ± 0.023	0.049	0.011	0.56	0.0035	0.87	1.7 e-08

*P*_T_ = *P* value of *t* test; *P*_U_ = *P* value of Mann–Whitney *U* test; *P*_S_ = *P* value of Spearman rank correlation.

Groups are mean ± SD.

### Microparametric Quantification of Bone Marrow

**FIGURE 4.**
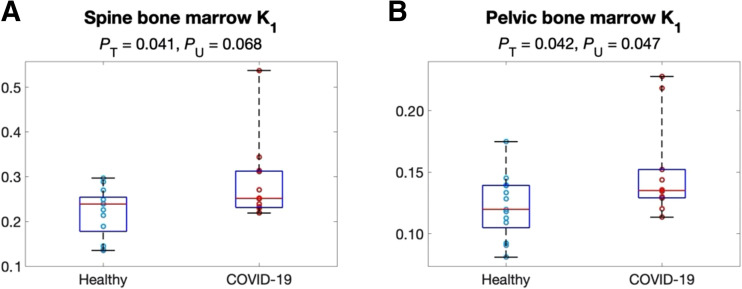
Comparison of $K_{1}$ of spine bone marrow (A) and pelvic bone marrow (B) between the healthy and the recovering COVID-19 groups. *P*_T_ = *P* value of *t* test; *P*_U_ = *P* value of Mann–Whitney *U* test.

The microparametric quantification results for bone marrow are summarized in Table 3. While bone marrow metabolism did not show a statistically significant difference between the 2 groups as measured with SUV, SUVR, or $K_{\text{i}}$ (Table 1), bone marrow $K_{1}$ was approximately 20% higher in the COVID-19 subjects with a statistical difference (*P* < 0.05), as shown in Figure 4 and Table 3. In comparison, no statistical significance was observed in $k_{2}$ or $k_{3}$. In contrast to the results in the lungs, the bone marrow microparameters $K_{1}$, $k_{2}$, and $k_{3}$ all had strong correlations with $K_{\text{i}}$, although the correlation of $K_{1}$ with $K_{\text{i}}$ remained relatively weak (Table 3).

**TABLE 3.** Comparison of Bone Marrow Microkinetic Parameters $K_{1}$, $k_{2}$, and $k_{3}$ Between Healthy Subjects and Recovering COVID-19 Subjects, and Correlation Between Microparameters and Bone Marrow $K_{\text{i}}$ Using Pearson and Spearman Analyses

Bone marrow type	Kinetic parameter	Comparison				Correlation with *K*_i_			
		Healthy group	Recovering COVID-19 group	*P*_T_	*P*_U_	Pearson		Spearman	
						*r*	*P*	ρ	*P*_S_
Spine	*K*_1_	0.221 ± 0.055	0.285 ± 0.089	0.041	0.068	0.46	0.020	0.39	0.056
	*k*_2_	0.76 ± 0.19	0.92 ± 0.31	0.14	0.20	0.45	0.023	0.35	0.091
	*k*_3_	0.0261 ± 0.0061	0.027 ± 0.013	0.73	0.76	0.78	3.5 e-06	0.82	2.2 e-06
Pelvic	*K*_1_	0.122 ± 0.026	0.149 ± 0.037	0.042	0.047	0.66	0.00032	0.71	9.5 e-05
	*k*_2_	0.573 ± 0.081	0.64 ± 0.14	0.17	0.26	0.51	0.0090	0.51	0.011
	*k*_3_	0.0246 ± 0.0060	0.0262 ± 0.0088	0.61	0.81	0.85	9.1 e-08	0.77	1.3 e-05

*P*_T_ = *P* value of *t* test; *P*_U_ = *P* value of Mann–Whitney *U* test; *P*_S_ = *P* value of Spearman rank correlation.

Groups are mean ± SD, *K*_1_ is in mL/min/cm^3^, and *k*_2_ and *k*_3_ are in min^−1^.

### Microparametric Quantification of the Spleen

**FIGURE 5.**
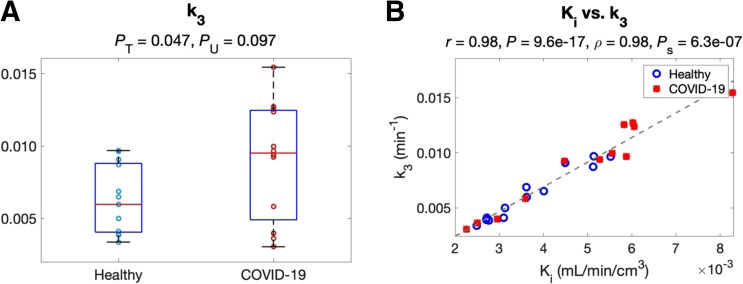
Study of microparametric quantification in spleen. (A) Comparison of $k_{3}$ between 2 groups. (B) Correlation between $k_{3}$ and $K_{\text{i}}$ among subjects. *P*_S_ = *P* value of Spearman rank correlation; *P*_T_ = *P* value of *t* test; *P*_U_ = *P* value of Mann–Whitney *U* test.

Table 4 shows the microparametric quantification results for the spleen. $k_{3}$ was approximately 45% higher in the COVID-19 group (Fig. 5A), whereas $K_{1}$ and $k_{2}$ did not show a significant group difference (*P* > 0.3). $k_{3}$ correlated the most strongly with $K_{\text{i}}$ among the 3 microparameters (Fig. 5B), indicating that the increased trend in spleen ^18^F-FDG metabolism (represented by SUVR and $K_{\text{i}}$) was dominated by increased phosphorylation. Overall, the observed changes in the spleen were similar to those of the lungs but with weaker statistical significance.

**TABLE 4.** Comparison of Spleen Microkinetic Parameters $K_{1}$, $k_{2}$, and $k_{3}$ Between Healthy Subjects and Recovering COVID-19 Subjects, and Correlation Between Microparameters and Spleen $K_{\text{i}}$ Using Pearson and Spearman Analyses

Kinetic parameter	Comparison				Correlation with *K*_i_			
	Healthy group	Recovering COVID-19 group	*P*_T_	*P*_U_	Pearson		Spearman	
					*r*	*P*	ρ	*P*_S_
*K*_1_ (mL/min/cm^3^)	1.61 ± 0.75	1.31 ± 0.88	0.37	0.40	−0.55	0.0044	−0.65	0.00052
*k*_2_ (min^−1^)	2.5 ± 1.0	2.1 ± 1.2	0.34	0.40	−0.43	0.034	−0.46	0.021
*k*_3_ (min^−1^)	0.0062 ± 0.0024	0.0090 ± 0.0041	0.047	0.097	0.98	9.6 e-17	0.98	6.3 e-07

*P*_T_ = *P* value of *t* test; *P*_U_ = *P* value of Mann–Whitney *U* test; *P*_S_ = *P* value of Spearman rank correlation.

Groups are mean ± SD.

### Effect of Vaccination

**FIGURE 6.**
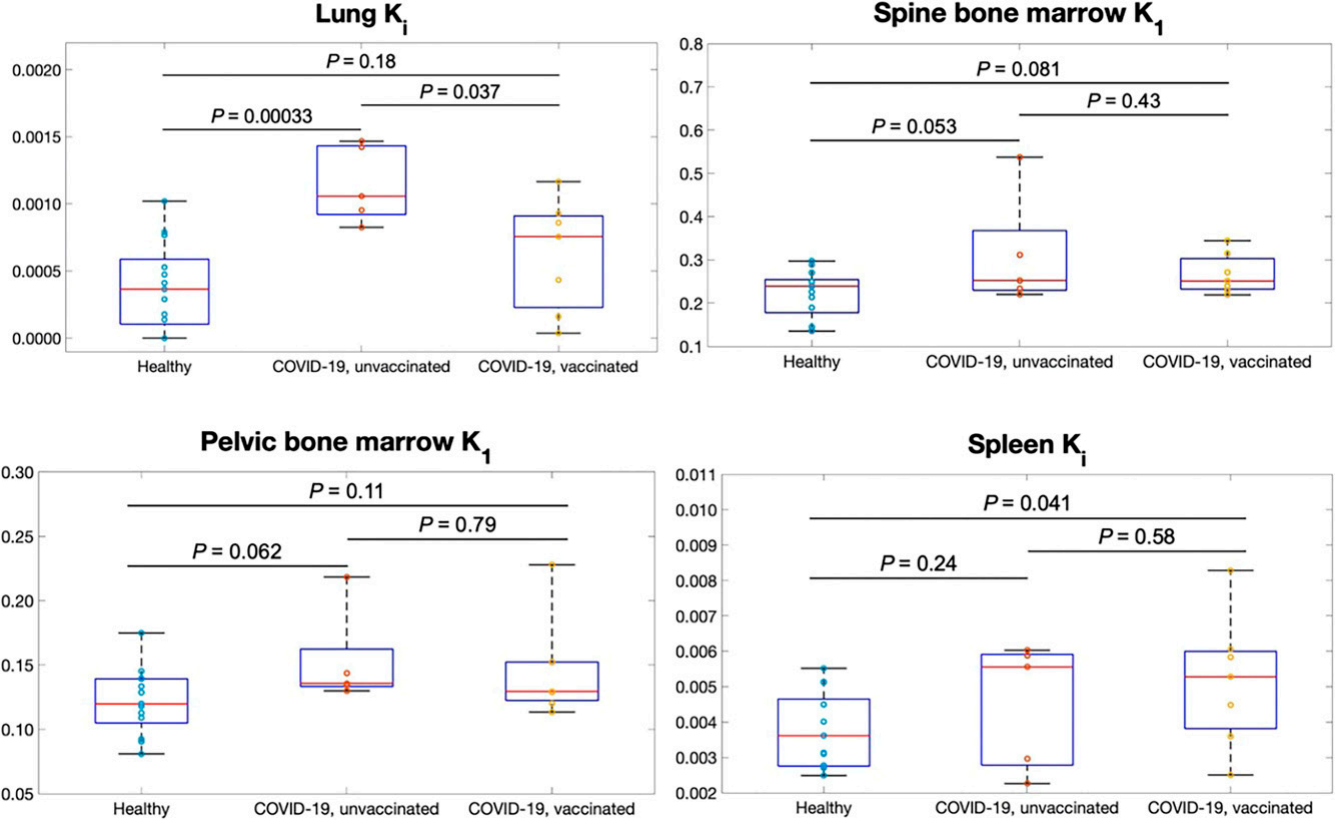
Evaluation of unvaccinated and vaccinated COVID-19 subjects compared with healthy subjects using kinetic parameters of interest: lung $K_{\text{i}}$, spine bone marrow $K_{1}$, pelvic bone marrow $K_{1}$, and spleen $K_{\text{i}}$. *P* values were calculated using unpaired *t* test.

Among the COVID-19 subjects, 5 subjects were unvaccinated and 7 subjects were vaccinated before their PET scans (Supplemental Table 1). There was no statistical difference in age, body mass index, or blood sugar level between the unvaccinated and the vaccinated COVID-19 subjects (*P* > 0.2). Lung $K_{\text{i}}$ was higher in unvaccinated COVID-19 subjects than in healthy subjects (*P* < 0.001), as shown in Figure 6. Lung $K_{\text{i}}$ was reduced in vaccinated COVID-19 subjects but still slightly higher than in the healthy group. Spine bone marrow $K_{1}$ of both unvaccinated and vaccinated COVID-19 subjects was higher than that of healthy subjects, but it differed little between unvaccinated and vaccinated COVID-19 subjects. Figure 6 also shows that the spleen $K_{\text{i}}$ of the vaccinated subjects tended to have a larger difference from the healthy subjects than the spleen $K_{\text{i}}$ of the unvaccinated ones. No effect of vaccination was noted in other organs of recovering COVID-19 subjects.

### Parametric Imaging of Recovering COVID-19 Subjects

**FIGURE 7.**
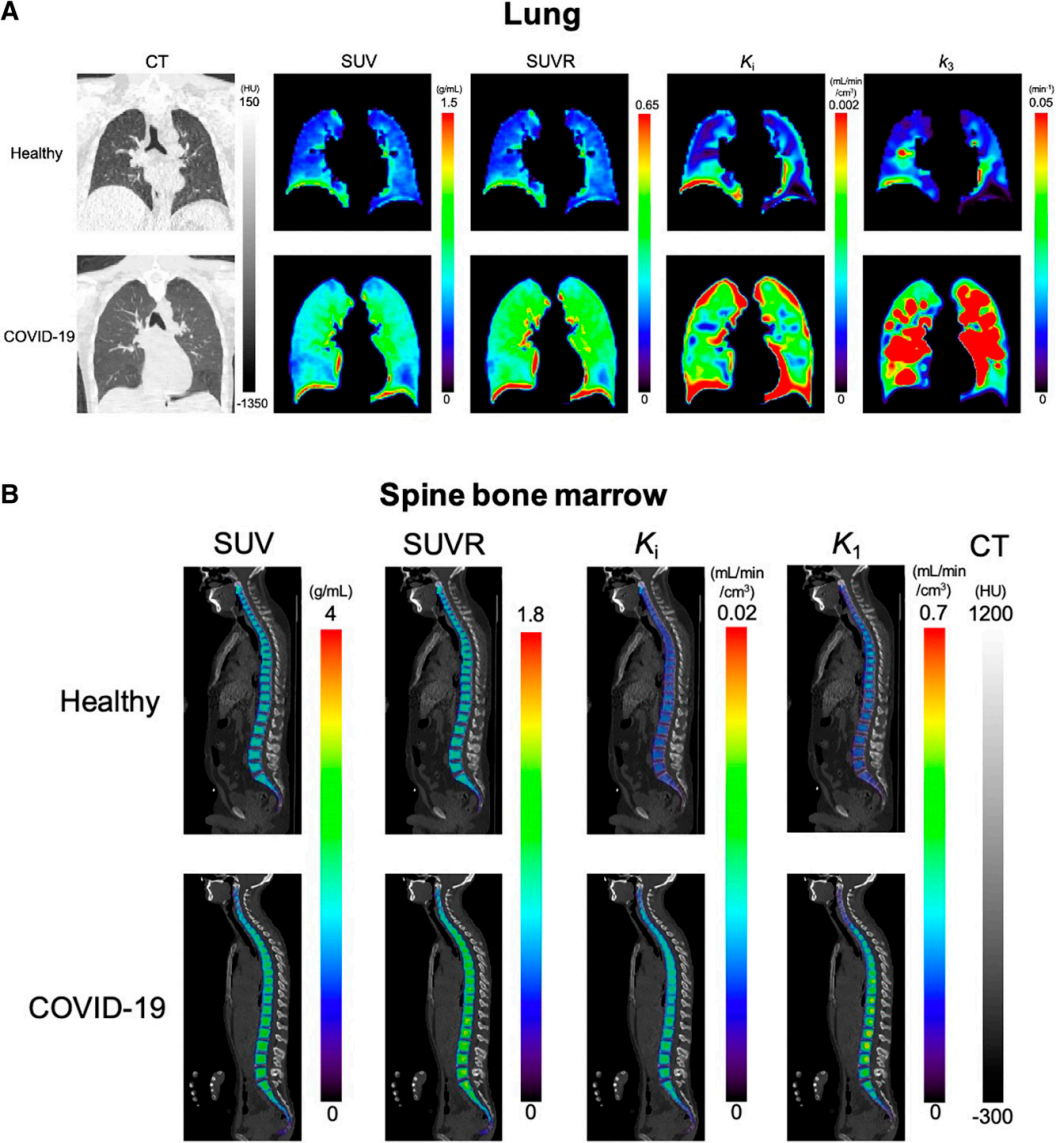
Parametric images of example healthy subjects and COVID-19 subjects. (A) Lung CT, ^18^F-FDG SUV, SUVR, and parametric images of $K_{\text{i}}$ and $k_{3}$. Coronal slices are selected as middle of trachea carina. (B) Spine bone marrow images of ^18^F-FDG SUV, SUVR, and parametric image $K_{\text{i}}$ and $K_{1}$. PET images are masked for bone marrow region and overlaid on CT images. HU = Hounsfield unit.

Figure 7 shows the parametric images of the lungs and bone marrow from healthy subjects and COVID-19 subjects. The lung images of SUVR, $K_{\text{i}}$, and $k_{3}$ showed enhanced contrast between the healthy and the recovering COVID-19 subjects compared with SUV (Fig. 7A) through visual inspection, supporting the ROI-based analyses. The demonstrated spatial heterogeneity across different lung lobes (Fig. 7A) is consistent with the lobe-based results of lung SUV and $K_{\text{i}}$, as reported in Supplemental Figure 4. In all 5 individual lung lobes, $K_{\text{i}}$ produced a larger statistical group difference than SUV.

The spine bone marrow (Fig. 7B) and pelvic bone marrow (Supplemental Fig. 5A) images of $K_{\text{i}}$ and $K_{1}$ showed increased contrast between the 2 subjects compared with SUV. The SUVR and $K_{\text{i}}$ images of the spleen also tended to have higher contrast than the SUV images (Supplemental Fig. 5B). These observations are consistent with the ROI-based findings.

## DISCUSSION

In this pilot study, we evaluated the metabolic differences in multiple organs between recovering COVID-19 subjects and healthy subjects using total-body dynamic ^18^F-FDG PET combined with kinetic modeling. This article focuses on establishing the technical foundation for quantitative measurements of glucose metabolism using total-body dynamic PET within the context of COVID-19, which helps inform and guide future research that involves subtle systemic changes, such as longitudinal tracking of long COVID-19.

We detected increased metabolism using $K_{\text{i}}$ in the lungs, whereas SUV or CT values gave no group differentiation (Table 1; Fig. 2), indicating the ability of lung $K_{\text{i}}$ to detect a subtle difference that is undetectable with SUV or CT. The inability of SUV to distinguish the groups likely occurs because of its semiquantitative nature and because it is susceptible to confounding factors (26). The results suggest the power of kinetic quantification for assessing glucose metabolism. The increased lung metabolism in the COVID-19 group may indicate continued inflammation during the early stages of recovery. Previous dynamic lung ^18^F-FDG PET studies have associated increased lung $K_{\text{i}}$ with pulmonary inflammation in multiple conditions, such as acute lung injury (33) and chronic obstructive pulmonary disease (34). Meanwhile, prolonged lung inflammation caused by COVID-19 has been reported; it can last more than 60 d after infection, even for asymptomatic patients and those with mild cases (35,36). The detected difference in lung glucose metabolism might potentially be related to the increased metabolism of immune cells, such as neutrophils (33,37,38) and macrophages (39,40), because of their accumulation and activation in the lungs.

Another advantage of compartmental modeling is microparametric quantification. According to the analysis in the lungs, $k_{3}$ is the parameter that was responsible for the healthy versus COVID-19 group difference in $K_{\text{i}}$ (Figs. 3 and 7A) and correlated best with $K_{\text{i}}$ among different microparameters (Table 2). The result implies that increased glucose phosphorylation, rather than glucose delivery, may be the main driving factor for increased lung metabolism. These findings are consistent with previous animal studies that observed $k_{3}$ increases in lung inflammation and the association between $K_{\text{i}}$ and $k_{3}$ (31*–*33,41).

Bone marrow demonstrated a significant change of $K_{1}$ in the recovering COVID-19 group compared with healthy subjects (Figs. 4 and 7B), but no differences were observed with SUV, SUVR, or $K_{\text{i}}$ that reflect overall ^18^F-FDG metabolism (Table 1). This result indicates the substantial importance of microparametric quantification. Bone marrow is essential for immunoregulation and is the origin of immune cells (42). Animal studies have reported that bone marrow cells play an important role in the repair of the injured lung during lung inflammation (43,44). Hence, the increased ^18^F-FDG delivery represented by $K_{1}$ may be associated with immune system response during COVID-19 recovery. Given that ^18^F-FDG $K_{1}$ of liver was also demonstrated to associate with hepatic inflammation in fatty liver disease (9,45), the interplay between $K_{1}$ and inflammation reaction and the potential of $K_{1}$ as a biomarker of disease are worth more studies to explore clinical applications.

The spleen tended to have higher glucose metabolism in the COVID-19 group, as represented by $K_{\text{i}}$ or SUVR (Table 1). This observation is consistent with the splenic ^18^F-FDG uptake increase reported in previous studies of COVID-19 (14) and other infectious diseases (46). As an immune organ, the spleen plays an important role in response to COVID-19 (47), and the immune response may lead to increased metabolism.

Our study also separated the unvaccinated and vaccinated COVID-19 groups to evaluate the potential effect of vaccination. The results from the unvaccinated COVID-19 subjects alone (Fig. 6) confirmed that COVID-19 is likely responsible for the observed differences in the lungs and bone marrow between the recovering COVID-19 group and the healthy group. Nonetheless, vaccination showed a combined effect on top of the impact of COVID-19. The lower lung $K_{\text{i}}$ in the vaccinated group may indicate reduced lung inflammation because of a protecting effect of vaccination. The higher spleen $K_{\text{i}}$ in the vaccinated subjects (Fig. 6) could also suggest increased immune response because of vaccination. These results are complicated by different vaccination conditions, such as the type, dose, and vaccination date before the PET scan.

This work has several limitations. First, the pilot study cohort is relatively small, especially in the comparison of unvaccinated (5 subjects) versus vaccinated (7 subjects). Therefore, the results, particularly concerning physiologic insights, should be interpreted with caution and warrant confirmation with future hypothesis-driven studies. With an increased sample size, it may be possible to observe some group differences that were not statistically significant in the current study. Second, the healthy and the COVID-19 groups are not exactly matched in this pilot study. Although there is no statistical difference in age, weight, body mass index, or blood sugar level between healthy subjects and recovering COVID-19 subjects, the unpaired age and the time variability between the COVID-19 diagnosis and the PET/CT scan could introduce potential bias. The percentage of women is higher in the COVID-19 group and further separated the analyses according to sex. Example results for lung SUV and $K_{\text{i}}$ are provided in Supplemental Figure 6 to indicate that the major findings of this work remained valid, although the statistical difference of $K_{\text{i}}$ became lower, primarily because of the limited sample size. Third, the study lacks histopathology or clinical laboratory data to elaborate on the reason for the differences in ^18^F-FDG kinetics between the 2 groups, and the potential impact of COVID-19 treatment on PET quantification was not analyzed because of the inaccessibility of medical records. In addition, some of the healthy cohort, although recruited between May 2019 and January 2020, before the COVID-19 pandemic (the first confirmed U.S. case was January 18, 2020), might have been exposed to COVID-19. Fourth, the statistical analysis in this pilot study was not corrected for possible familywise error rate, because the focus of this work is on comparing parametric metrics with SUV. Confirmation of the physiologic findings from this study will require a larger sample size with an appropriate correction for multiple comparisons. Finally, the kinetic model for ROI-based analysis and parametric imaging (31,32) used in this work followed a commonly used 2-tissue model for analyzing ^18^F-FDG data and considered time delay and organ-specific input functions. More advanced and organ-specific compartmental models could be investigated, for example, the 3-tissue model (33) and the recent high-temporal resolution model (48) for the lungs. We are investigating such models.

Our next steps are to use a similar methodology and more advanced models to study the impact of long COVID-19 on individual subjects. The interplay and correlation of tracer kinetics among different organs will be of interest. In addition, the results from this pilot work suggest future study designs should focus more on immune-related metabolic changes, for example, by tracking macrophage (49) or neutrophil (50) recruitment or monitoring serum inflammatory factors, to gain a deeper understanding of the prolonged impact of COVID-19 on glucose metabolism.

## CONCLUSION

With total-body multiparametric PET, increased lung ^18^F-FDG metabolism (measured by $K_{\text{i}}$) and increased bone marrow ^18^F-FDG delivery (measured by $K_{1}$) were detected in recovering COVID-19 subjects compared with healthy subjects. The changes may be associated with continued inflammation and immune response during the early stages of recovery from COVID-19. Vaccination may have a protection effect. These findings are missed or not possible to find if standard SUV measures are used. Total-body multiparametric ^18^F-FDG PET can be a more sensitive tool than conventional whole-body static ^18^F-FDG imaging for detecting subtle changes and may be used to study postacute sequelae of COVID-19.

## DISCLOSURE

This research is supported in part by National Institutes of Health grants R01 CA206187, R01 DK124803, and R01 AR076088. University of California, Davis, has a research agreement and revenue-sharing agreement with United Imaging Healthcare. No other potential conflict of interest relevant to this article was reported.

## Notes

KEY POINTS **QUESTION:** Compared with standard whole-body ^18^F-FDG PET imaging, is there benefit from using total-body multiparametric ^18^F-FDG PET to study COVID-19 recovery? **PERTINENT FINDINGS:** Higher ^18^F-FDG net influx and phosphorylation in the lungs and higher ^18^F-FDG blood-to-tissue delivery in bone marrow were detected in recovering COVID-19 subjects than in healthy subjects, whereas no statistical difference was detected using SUV. **IMPLICATIONS FOR PATIENT CARE:** Total-body multiparametric ^18^F-FDG PET may offer a more sensitive tool than SUV for quantitative assessment of multiorgan effects in COVID-19 recovery and may be used to study long COVID-19.
